# A rapid genome-wide response to *Drosophila melanogaster *social interactions

**DOI:** 10.1186/1471-2164-8-288

**Published:** 2007-08-22

**Authors:** Ginger E Carney

**Affiliations:** 1 Department of Biology, Texas A&M University, College Station, TX, USA

## Abstract

**Background:**

The actions and reactions integral to mate recognition and reproduction are examples of multifaceted behaviors for which we are only beginning to comprehend the underlying genetic and molecular complexity. I hypothesized that social interactions, such as those involved in reproductive behaviors, would lead to immediate and assayable changes in gene expression. Such changes may have important effects on individual reproductive success and fitness through alterations in physiology or via short-term or long-term changes in nervous system function.

**Results:**

I used Affymetrix *Drosophila* Genome arrays to identify genes whose expression profiles would change rapidly due to the social interactions occurring during *Drosophila melanogaster *courtship. I identified 43 loci with significant expression profile changes during a 5-min exposure period. These results indicate that social interactions can lead to extremely rapid changes in mRNA abundance.

**Conclusion:**

The known functions of the up-regulated genes identified in this study include nervous system signaling and spermatogenesis, while the majority of down-regulated loci are implicated in immune signaling. Expression of two of the up-regulated genes, *Odorant-binding protein 99b (Obp99b) *and *female-specific independent of transformer (fit)*, is controlled by the *Drosophila* sex-determination gene hierarchy, which regulates male and female mating behaviors and somatic differentiation. Therefore, additional identified loci may represent other long-elusive targets of *Drosophila* sex-determination genes.

## Background

Social interactions are known to alter individual physiology, but the extent to which transcriptional or post-transcriptional mechanisms function in inducing these changes is unclear. Immediate responses are likely modulated by environmentally induced changes in synaptic signaling, which may result in alterations in gene expression with long-term consequences. Identifying the genetic basis for physiologic responses is of significant interest because it will clarify how regulated gene expression and the resulting intracellular signaling events modulate complex behaviors.

Genes that control complex traits such as behaviors are difficult to identify by standard mutant analysis for two major reasons: (1) behaviors may be influenced by a large number of genes which each have a small effect, and (2) the genes involved usually have pleiotropic functions dependent upon the cellular or developmental context. Despite these limitations, *Drosophila* geneticists have successfully used mutant analysis to identify loci integral to behaviors such as learning and memory, circadian rhythmicity, foraging and reproduction [Reviewed in [1]].

Newer technologies are also useful for gene discovery. Genomics approaches are widely used for identifying candidate behavior gene loci [2-9] that can be experimentally validated using other approaches. High throughput RNAi screens in cultured *Drosophila* cells have been used to identify genes involved in numerous cellular processes [Reviewed in [10]], and in vivo RNAi-based screens are now possible because of the recent creation of an RNAi *Drosophila* strain library [11]. These RNAi strains can be used to identify behavior genes by targeting specific cells, tissues and developmental periods.

Courtship behaviors in *Drosophila* are genetically programmed and require multi-modal inputs and outputs. Male flies use sight, smell, taste, and touch to identify appropriate mates [Reviewed in [12] and [13]] and learn to distinguish a conspecific female from a heterospecific female [14]. Females initially respond to male courtship advances by running away, but a female that receives and responds to appropriate signals from a courting male will allow herself to be mated, usually within 5–10 min.

Several major regulatory genes for *D. melanogaster *courtship and reproduction have been identified [Reviewed in [12,13,15-17]], but only a small number of potential downstream targets are known [18-25]. The genes controlling reproduction as well as other behaviors can function during different time periods. Some of them may be required for development and differentiation of a sex-specific neural circuit. Recent work has placed *fruitless (fru) *into this category of genes [26-29]. The male-specific Fru protein directs development of a nervous system with male-specific neuron numbers and projections [27].

Other gene products may direct the function of the circuit by ensuring that the animal is primed to execute a behavior in response to environmental cues. *fru *also functions in this manner since inactivating *fru *neurons in adult males inhibits courtship behavior [28]. Finally, a third class that I refer to as "effector" genes should respond to the initiation or performance of a behavior to generate short-term and long-term changes in nervous system function and animal physiology. Genes in this group are expected to change expression as a consequence of behavioral responses. I hypothesized that loci in these three categories would be identifiable via whole genome assays of animals that recently performed a behavior of interest.

A recent microarray study in *Drosophila* suggested that there are differences in gene expression patterns between unexposed and courted (but unmated) females two hours after male exposure, indicating that the females respond at the genetic level to courtship [30]. Females also respond genetically to mating and the transferred male ejaculate [30,31]. Because courtship and mating occur quickly in *Drosophila melanogaster*, I reasoned that measurable changes in gene expression likely occur on a rapid timescale in flies as a consequence of pre-mating interactions. I tested this hypothesis by allowing virgin male flies to court, but not mate, virgin female flies during a 5-min interval and collecting whole-body RNA from the males to assay mRNA levels using Affymetrix *Drosophila* Genome Arrays. The results indicate that a small number of loci rapidly respond to these pre-mating interactions.

## Results

### Courtship interactions cause rapid changes in mRNA abundance

I exposed *Drosophila melanogaster *males to conspecific female courtship objects and tested male flies for rapid gene expression changes resulting from this social interaction. Single, four-day-old virgin male flies were allowed to court a virgin female for 5-min (See Methods). Only males that courted the female but did not mate during this time period were collected for RNA processing and microarray hybridization. The *Drosophila* Genome Arrays (Affymetrix version 1) used in these experiments contain probe sets for over 13,500 predicted transcripts based upon annotation of the *Drosophila* genome.

I used three data extraction methods (See Methods) to derive expression values that could be compared for statistically significant differences (I compared chips hybridized to labeled RNA from unexposed males to chips hybridized with labeled RNA from courting males). Only those genes statistically significant (p < 0.001) for at least two of the three data extraction methods are shown. The final data set comprises 43 genes with altered expression levels due to courtship interactions (Tables 1 and 2). Ten genes are up regulated, and 33 genes are down regulated based upon the described criteria. The majority of genes in the data set have p-values that are well below this cutoff (Tables 1 and 2).

**Table 1:** Genes up regulated in courting males

**Gene identifier**	**Gene name**	**Avg. fold change**	**GCOS p-value**	**PM+MM p-value**	**PM only p-value**
CG6128		2.58	3.48E-04	1.27E-04	1.93E-05
CG13155		2.26		1.46E-04	6.27E-05
CG7738		2	7.57E-04	1.09E-06	3.22E-06
CG1732		1.89	7.29E-04	3.57E-05	1.82E-04
CG33060		1.85	1.41E-04	1.35E-05	4.65E-05
CG30042		1.8	9.40E-04	1.63E-05	5.21E-04
CG2958	*lectin-24Db*	1.71		5.29E-04	6.01E-04
CG17820	*fit*	1.6	5.70E-04	7.20E-05	1.10E-04
CG7592	*Obp99b*	1.52	2.44E-04		7.17E-04
CG7106	*lectin-28C*	1.48	6.77E-04	3.46E-04	2.11E-04

Ten genes are significantly (p < 0.001) up regulated in males exposed to a conspecific female courtship object. The average fold change from microarray analysis is shown. The p-values are from statistical analyses performed on expression values obtained by three different methods (See Methods).

**Table 2:** Genes down regulated in courting males

**Gene identifier**	**Gene name**	**Avg. fold change**	**GCOS p-value**	**PM+MM p-value**	**PM only p-value**
CG6639		-21.2	1.12E-10	3.17E-10	1.24E-11
CG4740	*AttC*	-11.79	3.00E-07	2.82E-05	4.02E-04
CG18372	*AttB*	-9.62		3.19E-04	4.94E-04
CG10146	*AttA*	-8.51	7.23E-04	3.80E-04	6.72E-04
CG4757		-7.69	1.26E-07	1.17E-06	1.65E-06
CG15066	*IM23*	-5.17	1.11E-08	1.13E-10	1.61E-10
CG8175	*Mtk*	-4.37	4.01E-06	1.88E-05	4.26E-05
CG1367	*CecA2*	-4.31	8.34E-04	1.06E-05	9.88E-04
CG13422		-4	3.16E-08	2.34E-07	3.71E-07
CG10810	*Drs*	-3.99	8.60E-07	7.77E-09	6.18E-09
CG10816	*Dro*	-3.3	2.73E-05	1.50E-08	3.13E-05
CG18563		-3.28	8.55E-06	1.57E-04	8.55E-06
CG1365	*CecA1*	-3.12	8.19E-04	4.65E-04	
CG6687		-2.84	2.39E-04	4.02E-05	6.84E-05
CG18108	*IM1*	-2.46	8.67E-08	3.41E-07	1.58E-07
CG9989		-2.18		1.00E-04	7.14E-04
CG5550		-1.93	1.12E-04	1.26E-04	2.18E-05
CG2217		-1.92		1.87E-05	2.42E-05
CG15065		-1.9	4.43E-05	9.75E-08	7.54E-08
CG14745	*PGRP-SC2*	-1.89		4.30E-06	6.96E-05
CG9434	*Fst*	-1.76		3.46E-04	1.29E-04
CG2042		-1.67		1.40E-04	8.27E-05
CG16772		-1.66	5.99E-04	3.71E-05	2.53E-05
CG13947		-1.57	2.79E-04	9.57E-05	7.27E-05
CG18279	*IM10*	-1.55	1.00E-03	2.06E-05	3.48E-05
CG13482		-1.48		3.75E-04	2.94E-04
CG18106	*IM2*	-1.42	5.99E-04		9.62E-04
CG11992	*Rel*	-1.42		3.12E-04	4.54E-04
CG16844	*IM3*	-1.4		3.07E-06	7.87E-07
CG15231	*IM4*	-1.28		9.52E-05	4.92E-06
CG18067		-1.27		1.89E-05	9.24E-06
CG10332/IM18		-1.05		3.66E-04	3.51E-04
CG10947		-1.05		2.74E-06	4.20E-05

Thirty-three genes are significantly (p < 0.001) down regulated in males exposed to a conspecific female courtship object. See the legend from Table 1 and Methods for a description of how the p-values were obtained. *CG10332 *and *IM18 *are overlapping transcription units that cannot be distinguished with the probe set on the Drosophila version 1 Genechip.

### Validation of array results

I chose a group of 4 up-regulated candidates and 9 down-regulated candidates for further validation by Real-time PCR analysis (Table 3) and prepared cDNA from the RNA used for microarray hybridizations as well as from independently obtained samples. Relative expression levels from Real-time PCR were calculated separately for microarray samples and the independent samples. In each instance, genes with altered expression levels according to the microarray analysis also showed the same directional change when the array samples were re-tested by Real-time PCR (Table 3).

**Table 3:** Real-time PCR validation of microarray results

**Gene identifier**	**Gene name**	**Array fold change**	**Array RT-PCR**	**Independent RT-PCR**
CG1732		1.89	2.94	2.86
CG17820	*fit*	1.6	2.48	1.25
CG7592	*Obp99b*	1.52	1.51	1.35
CG7106	*lectin-28C*	1.48	1.2	1.92
CG16834	*lectin-33a*	1.87	5.37	4.91
				
CG4740	*AttC*	-11.79	-19.26	-3.01
CG4757		-7.69	-3.73	1.07
CG15066	*IM23*	-5.17	-6.46	-1.07
CG8175	*Mtk*	-4.37	-2.41	1.01
CG1367	*CecA2*	-4.31	-3.75	-1.04
CG13422		-4	-5.25	2.11
CG10816	*Dro*	-3.3	-40.05	-1.33
CG6687		-2.84	-2.82	1.07
CG9434	*Fst*	-1.76	-2.79	1.6

Five genes up regulated in courting males were verified by Real-time PCR on the samples used for array hybridization (Array RT-PCR) as well as by analysis of independently obtained samples (Independent RT-PCR). Nine down-regulated genes were validated using array samples, while 4 of 9 were validated on independent samples.

Real-time PCR amplification from independent samples verified the increased expression of all four tested genes (*CG1732*, *fit*, *Obp99b*, *lectin-28C*) that were up regulated in exposed males according to the microarray analysis. The expression differences for *fit *and *Obp99b *are statistically significant between the two treatments (Table 4).

**Table 4:** Relative expression levels of up-regulated candidate genes

**Gene identifier**	**Gene name**	**Unexposed males (± SEM)**	**Courtship-exposed males (± SEM)**
CG1732		0.12 ± 0.02	0.32 ± 0.1
CG17820*	fit*	3.15 ± 0.53	5.21 ± 0.6
CG7592*	Obp99b*	5.00 ± 0.75	7.00 ± 1.52
CG7106	*lectin-28C*	0.67 ± 0.08	0.90 ± 0.12
CG16834*	lectin-33a*	0.24 ± 0.1	1.25 ± 0.27

* indicates up-regulated genes with significantly different levels (p < 0.05) of expression when courtship-exposed and unexposed male samples are compared.

In contrast, not all of the down-regulated genes tested by Real-time PCR on independent samples showed the directional change predicted from the microarrays. However, a regression of mean expression fold-changes based on the microarray analysis versus mean fold-changes based on Real-time PCR of independently collected samples indicated a highly significant positive correlation between results obtained by the two methods (r = 0.74, N = 14, p = 0.003). The p-values are non-significant when the down-regulated and up-regulated genes are considered separately. I attribute this result to the small sample size for each category, particularly since the genes with increased expression show the same trend in all analyzed samples. Thus, even though there is some error associated with the assays (see Discussion), the Real-time PCR results strongly support the microarray results.

### Up-regulated genes

Genes with increased levels of expression due to reproductive interactions comprise a group with diverse functions as predicted by Gene Ontology (GO) annotations (Table 5). The two lectin genes, *lectin-24Db *and *lectin-28C*, are likely to function in spermatogenesis. A third lectin gene, *lectin-33a*, was identified as significantly up regulated (1.87-fold) only in the dChip PM-MM analysis, so it did not make the cut off. However, Real-time PCR confirms that *lectin-33a *is up regulated approximately 5-fold on average in the array and independent samples (Tables 3 and 4).

**Table 5:** Predicted functions of up-regulated genes

**Gene identifier**	**Gene name**	**GO molecular function**	**GO biological process**	**Immune response**
CG6128		alpha-L-fucosidase	O-glycoside catabolism; fucose metabolism	n
CG13155		unknown	unknown	n
CG7738		unknown	unknown	y
CG1732		GABA:sodium symporter activity	ion transport	n
CG33060		Unknown function	unknown	n
CG30042		unknown	unknown	n
CG2958	*lectin-24Db*	galactose, fucose, mannose binding	spermatogenesis	n
CG17820	*fit*	unknown	unknown	n
CG7592	*Obp99b*	odorant binding	autophagic cell death/olfactory behavior	n
CG7106	*lectin-28C*	galactose binding	spermatogenesis	n

Most intriguingly, two previously identified targets of the sex-determination hierarchy, *Obp99b *and *fit *[22], are up regulated in exposed males. *Obp99b *(also known as *tsx*) encodes an odorant binding protein that is enriched in adult male heads [5,22] and is regulated by the sex-determination hierarchy genes, *Sex-lethal (Sxl), transformer (tra)*, *transformer-2 (tra-2) *and *doublesex (dsx) *[22]. Although the specific functions of odorant binding proteins (Obps) are controversial, one possibility is that Obps are secreted into the olfactory sensillum and bind to and transport odorants to odorant receptors for downstream signal transduction [Reviewed in [32]]. Therefore, Obps may function in modulating olfactory responses to courtship.

### Down-regulated genes

Using the Gene Ontology database information available through Flybase, I determined the likely function of the genes with decreased expression in exposed males relative to unexposed controls (Table 6). Twenty-seven out of 33 genes with reduced abundance are loci previously implicated in innate immunity [33-37]. The functions of the remaining 6 genes are not known. Decreased expression of some immune response genes was supported by Real-time PCR (Table 3); however *CG4757 *was the only down-regulated gene with a statistically significant difference in expression levels between the two treatments (See Methods). Given the general trend for immunity gene expression to be less abundant in courting males (Table 2) and previous observations indicating that mated males have decreased immunity [38], I hypothesize that a general but non-specific decrease in levels of immune response genes occurs in courting males.

**Table 6:** Predicted functions of down-regulated genes

**Gene identifier**	**Gene name**	**Location**	**GO molecular function**	**GO biological process**	**Immune response**
CG6639		36C9	chymotrypsin activity	proteolysis	y
CG4740	*AttC*	50A3	unknown	antibacterial humoral response	y
CG18372	*AttB*	51C1	unknown	antibacterial humoral response	y
CG10146	*AttA*	51C1	unknown	antibacterial humoral response	y
CG4757		86D5	carboxylesterase	unknown	y
CG15066	*IM23*	55C4	unknown	antibacterial humoral response	y
CG8175	*Mtk*	52A1	unknown	antibacterial/antifungal humoral response	y
CG1367	*CecA2*	99E2	unknown	antibacterial humoral response	y
CG13422		57A4	glucosidase	antibacterial defense response	y
CG10810	*Drs*	63D2	ion channel inhibitor	antibacterial/antifungal humoral response	y
CG10816	*Dro*	51C1	unknown	antibacterial humoral response	y
CG18563		36C9	trypsin activity	proteolysis	y
CG1365	*CecA1*	99E2	unknown	antibacterial humoral response	y
CG6687		88E3	serine-type endopeptidase inhibitor; ATP synthase	ATP synthesis coupled protein transport; proteolysis	y
CG18108	*IM1*	55C4	unknown	defense response	y
CG9989		98E1	unknown	unknown	n
CG5550		53D10	receptor binding	defense response	y
CG2217		99F3-4	unknown	unknown	n
CG15065		55C4	unknown	unknown	y
CG14745	*PGRP-SC2*	44E2	peptidoglycan binding	defense response	y
CG9434	*Fst*	85E2	unknown	response to cold	y
CG2042		39D1	unknown	unknown	n
CG16772		38A8	unknown	unknown	n
CG13947		21E2	unknown	unknown	n
CG18279	*IM10*	50A5	unknown	antibacterial humoral response	y
CG13482		70D5	unknown	unknown	y
CG18106	*IM2*	55C4	unknown	defense response	y
CG11992	*Rel*	85C3	transcription factor	defense response	y
CG16844	*IM3*	55C4	unknown	antibacterial humoral response	y
CG15231	*IM4*	57B3	unknown	defense response	y
CG18067		57A5	unknown	unknown	y
CG10332/IM18		59F4	unknown	defense response	y
CG10947		38C6	unknown	unknown	n

Twenty-seven of the 33 down-regulated genes likely function in the immune response. *CG15065 *is closely related to *IM2*. Immune responsive genes are overrepresented in this data set (p = 3.163 × 10^-32^, Fisher's Exact Test).

## Discussion

These findings indicate that short-term social interactions between flies can lead to rapid genetic responses. Two recent reports support the possibility of rapid changes in mRNA abundance, even within a 5-min interval. Yeast increases expression of approximately 2500 genes within 6 min of a change in environmental conditions [39]. Such rapid changes in mRNA abundance are not limited to single-celled organisms with small genes. The cichlid fish *Astatotilapia burtoni *can ascend to social dominance within minutes of being provided with environmental conditions that effect this change [40]. The transcription factor *egr-1 *is rapidly induced in the brains of these fishes within a similarly short timeframe and may regulate genes that mediate long-term physiological effects of social dominance [40]. Other studies have shown that longer periods of behavioral stimulation can lead to changes in gene expression [41,42], some of which are detected even 24 hr post-exposure [6,43,44].

How are rapid changes in gene expression regulated? It is likely that transcriptional as well as post-transcriptional mechanisms are involved. In the case of stationary phase yeast, RNA Pol II appears to be located at the promoters for genes that are rapidly induced by changes in environmental conditions [39]. For metazoans such as flies, a similar regulatory mechanism can be used [Reviewed in [45]]. RNA Pol II pauses on the *hsp70 *promoter prior to heat-shock induction [46], and heat shock induces *hsp70 *expression within 30–60 sec [47]. Heat shock also rapidly inhibits transcription of the histone *H1 *gene [47].

Another possibility is that the increases or decreases in transcript abundance are due to post-transcriptional controls. Cells may respond to environmental stimuli by altering degradation patterns of specific transcripts, increasing or decreasing them as needed. Post-translational regulation of proteins via phosphorylation, for instance, may also account for part of the rapid response. *Drosophila* immune response genes are induced rapidly after a pathogen assault via post-translational activation of NF-κB transcription factors [See ref [48]].

I identified a set of 43 loci with immediate alterations in mRNA abundance in male flies as a consequence of 5-min courtship interactions. Four of 10 up-regulated genes were retested and verified by Real-time PCR [Tables 3 and 4]. The up-regulated genes are involved in processes likely to be important for mating and reproduction such as neurotransmission (*CG1732 *and *Obp99b*) and spermatogenesis (*lectin-24Db *and *lectin-28C*). The three lectin genes are unique at the nucleotide level, so cross hybridization is unlikely to account for the fact that these genes were identified in my analysis. Lectin-24Db and Lectin-28C are possible paralogs of the accessory gland protein Acp29Ab [49] that is transferred to females via the male ejaculate. Male flies, once they encounter a potential mate, may prepare to increase sperm and Acp production in anticipation of an imminent mating that will deplete ejaculate components.

None of the up-regulated candidates are known to interact with one another genetically or via protein-protein interactions, although 3 of the genes (*CG13155*, *CG7738*, *lectin-33a*) have predicted interaction partners from a yeast two-hybrid screen [50]. Only *CG6128 *has available alleles within the gene; both are P-element insertions within a very large upstream intron. Animals homozygous for the insertions are viable and fertile, which likely indicates that the insertions do not affect gene function.

Some of the candidates with increased expression are enriched in adult head tissue [51]. As expected from previously reported results [22], *fit *and *Obp99b *are enriched in head tissue but not in the brain [51]. *CG1732 *is expressed in the adult head (including the brain) and male accessory glands. Intriguingly, *CG1732 *is also enriched in larval fat body. *CG7738 *is enriched in the head, while *CG33060 *is expressed in the testis [51]. These expression patterns are consistent with my hypothesis that the candidates regulate reproductive behavior or sperm production.

Of particular interest from this study are the two previously identified targets of the *Drosophila* sex-determination hierarchy, *Obp99b *and *fit*, which are sex-specifically expressed. The sex-determination genes control somatic sexual differentiation and sex-specific reproductive behaviors [Reviewed in [15]] and are expressed during development as well as in adult flies. *Sxl *regulates splicing of *tra*, which together with *tra-2*, directs sex-specific splicing of transcripts for *fru *and *dsx*. *fru *and *dsx *encode transcriptional activator proteins [52,53], but only a small number of targets for these factors have been identified [18-25]. *fru *is the major regulator of male sexual behavior [16] and directs male-specific neuronal development by regulating apoptosis as well as differentiation of neuronal processes [27,29,54]. Fru neuron function also is required in the adult male for numerous reproductive behaviors [28].

*Sxl*, *tra *and *tra-2 *regulate expression of *Obp99b *so that it is present at high levels only in males [22]. Loss-of-function mutations in *Obp99b *have not been reported, but females over expressing *Obp99b *do not mate at wild-type frequency [22], possibly indicating that *Obp99b *has a behavioral function.

The second sex-determination target identified in this experiment, *fit*, is enriched in female heads in the fat body surrounding the brain and is regulated by the sex-determination gene *Sxl *but does not require *tra *or *tra-2 *function [20]; potential regulation of *fit *expression in males by the sex-determination hierarchy has not been examined. *fit *expression varies depending upon the genetic background; it is expressed at low levels in males of the *w*^1118 ^and *tra-2/CyO *genotypes but is undetectable in *Oregon-R *[22]. Levels of *fit *are elevated 2 hrs post mating (G.E.C. and L.L. Ellis, unpublished results). However, two hours after a courtship exposure, males that do not mate have *fit *levels similar to those of unexposed animals (G.E.C. and L.L. Ellis, unpublished results).

Interestingly, *fit*, *Obp99b*, and a third sex-determination target gene, *takeout*, are expressed in the fat body cells surrounding the adult brain [21,22], and *Obp99b *and *takeout *are expressed in the chemosensory systems of both sexes [21,55]. *takeout *has a known role in reproductive behavior; feminization of male *takeout*-expressing cells results in reduced courtship [21], and over expression of *Obp99b *in females affects their latency to copulation [22]. My Real-time PCR results indicate that a male-enriched *takeout*-related gene, *CG5867 *[21], is also up regulated in courting males compared to unexposed males (data not shown). Together, these results suggest that *Obp99b *and *fit *also function in male reproduction and support previous work indicating that gene expression in the fat body of the head may have important functions in sex-specific reproductive behavior and physiology [21,22]. The fact that I identified at least two sex-determination target genes indicates that the expression changes are not simply due to differences in male activity levels.

I also report that 27 of 33 down-regulated genes are implicated in the innate immune response of flies. However, I was able to verify only a small number of the changes using independent samples (Table 3), and only one gene, *CG4757*, showed a statistically significant decrease in expression in courtship-exposed males (See Methods). While the trend indicates a general decrease in the transcript levels of immune response genes as a consequence of courtship interactions, the genes involved appear to be widely variable. Some down-regulated immunity genes are located at adjacent chromosome regions, possibly indicating that they are co-regulated. There are groups of immune genes at chromosome location 36C9 (*CG6639*, *CG18563*), 51C1 (*AttA*, *AttB*, *Dro*), 55C4 (*IM1*, *IM2*, *IM3*, *IM23*, *CG15065*) and 99E2 (*CecA1*, *CecA2*). The 55C region contains a head-specific cluster of co-regulated gene products [56]. The immune genes at 51C1, 55C4 and 99E2 share sequence similarity (as do *AttA *and *AttB *with *AttC*), so partial cross-hybridization to probe sets may account for correlations among hybridization signals.

One possibility is that the immune system is not down regulated in this behavioral paradigm, and the assays are simply capturing a snapshot of the natural variation in immune response gene expression. An alternative hypothesis is that there is more than one way to rapidly down regulate the energetically costly immune response system. In this scenario, the specific genes are unimportant as long as resources previously spent bolstering the immune response are directed toward ensuring reproductive success. Since earlier work showed that mated males have decreased immunity that is correlated with the number of matings [38], it is plausible that males begin down-regulating immune response genes once mating appears likely. It is worth noting that antimicrobial peptides, similarly to *Obp99b *and *fit*, are produced in the fat body, indicating that regulation of fat-body enriched transcripts throughout the body may play an important role in reproductive success.

Two other groups assayed the female genetic response to male courtship [30] or mating [30,31]. Somewhat surprisingly, both groups found that *fit *expression is increased in mated females [30,31]. In contrast to my results in males in which I find that immune response genes are generally down regulated, both groups observed increased expression of immune response loci in mated females [30,31].

## Conclusion

I found that flies can rapidly alter gene expression patterns in the context of social interactions that occur during reproduction. Such changes in mRNA abundance are likely significant to individual reproductive success. Since the present experiment measured changes occurring in the entire male body, I likely missed many subtle changes such as those occurring in the central nervous system (CNS) or other tissues in only a small number of cells. Future experiments will be designed to identify additional potential candidates in the CNS as well as to differentiate between genes that are specific to reproductive behavior and those involved in more general social interactions among flies.

## Methods

### Affymetrix Microarrays

I collected virgin, wild-type *Canton-S *males and females and aged them at 25° in groups of 20 or fewer flies. Males were transferred individually to new vials on day 3. On day 4 a single female was aspirated into a vial containing a single male, and the pairs were observed for 5 min at 22°. Males for the unexposed treatment were mock aspirated.

Thirty-five percent of the males displayed robust courtship without mating (these animals were collected for RNA preparation), and 36% mated within the 5 min observation period. The remaining males (29%) generally performed at least early courtship behaviors but did not attempt copulation. Males who demonstrated robust courtship toward the females (following, wing extension, copulation attempts) were collected, quick-frozen and stored at -80° for later RNA extraction. Only males who did not copulate during this 5-min window were collected. The time from completion of courtship to freezing was less than one minute for all samples.

Males were randomly pooled in groups of approximately 12 individuals, and total RNA was extracted in Trizol reagent (Invitrogen) following standard protocols. Control samples consisted of males that were treated identically except that they were not exposed to a courtship object. All collections and assays were performed at the same time each day to avoid circadian effects on gene expression.

Three RNA samples from males that courted females and three RNA samples from unexposed males were used to probe *Drosophila* Genome Arrays (Affymetrix version 1, based upon Berkeley *Drosophila* Genome Project v4.0) for a total of six arrays. RNA labeling and hybridization using standard Affymetrix protocols were performed at the University of Kentucky MicroArray Core Facility or at the Texas A&M University Department of Biology Core Facility.

The statistical analysis of the six arrays (three experimental and three control) roughly followed that of Lawniczak and Begun [30]. Expression values were calculated by using three different methods: the method implemented in the GCOS software package (Affymetrix), the PM-only method of dChip [57] and the PM-MM method of dChip [57]. PM refers to a perfect match between the probe sequence and the *Drosophila* reference sequence, while MM (mismatch) refers to a single nucleotide difference between the probe and reference sequence that should affect RNA hybridization to the probe.

Each of these three sets of expression values was then analyzed separately with the Bayesian t-test implemented in Cyber-T [58]. In this experiment, groups of experimental and control animals were collected and analyzed in parallel, so I used Cyber-T's paired t-test option to test the null hypotheses of no change in expression level between exposed and unexposed males for each transcript represented on the microarray. For a transcript to be included in the analysis, I required it to display an expression value of at least 100 on at least three of the six experimental and control arrays. Following Lawniczak and Begun [30], I used p < 0.001 as a significance threshold to reject the null hypothesis. In general, I considered a transcript to exhibit a significant change in expression level only if it fulfilled these analysis criteria in at least two of the three separate statistical analyses (i.e., GCOS, dChipPM-MM, dChipPM-only). See Lawniczak and Begun [30] for a much more detailed discussion of this approach.

### Real-time PCR

To validate the microarray results, the Superscript 1^st ^Strand Synthesis Kit (Invitrogen) was used to prepare cDNA from independently obtained RNA samples (3 independent experimental and 3 independent control preparations) as well as from two sets of samples used for microarray analyses. I did not have sufficient RNA remaining from the third array sample to use for cDNA preparation.

cDNA preparations were diluted 1:15, and 1.5 ul was used in each reaction for Real-time PCR using the SYBR Green PCR Mastermix (Applied Biosystems) essentially as described previously [59]. Reactions were performed in the ABI7700 (Applied Biosystems) using the default run parameters.

I chose five up-regulated candidates for validation; however, only four of the primer pairs generated gene-specific amplification products (*CG1732*, *fit*, *Obp99b*, and *lectin-28C*). Since most of the 33 down-regulated candidates are implicated in immune signaling, I chose a smaller number of candidates for PCR validation. I selected 9 genes that are not closely linked and are either uncharacterized or are known targets of each of the two major immune signaling gene cascades. Since Real-time PCR validated few of the down-regulated immune candidates, I did not continue to test additional genes from this group.

*rp49 *primers were used in control amplification reactions for normalizing the amount of cDNA in each preparation [59]. The Relative Standard Curve Method (Applied Biosystems) was used to determine relative levels of RNA for each sample. Values from female-exposed males were normalized to unexposed males to derive an average fold change in expression.

Each experiment included control reactions for each primer pair to test for amplification specificity in the presence or absence of template. A melting curve analysis was performed at the end of each run to test for primer specificity. As a second test for presence of the correct product, selected reactions were electrophoresed on agarose gels to view PCR amplification products.

I calculated the fold change for the Genechip hybridization samples independently from the three samples that were used only for Real-time PCR confirmation. All of the up-regulated genes tested had the expected directional increase. I grouped the 5 sets of samples together for statistical analysis in order to increase the sample size for the comparisons and performed a two-tailed t-test. Levels of *fit*, *Obp99b*, and *lectin-33a *and are significantly different (p < 0.05) between the unexposed and exposed treatments, and all 3 genes are up regulated by courtship exposure. While the levels of *CG1732 *and *lectin-28C *are consistently higher in courtship-exposed males compared to mock-exposed males, the two treatments are not statistically different from one another. I attribute this finding to the small sample size and the variation in the absolute levels of transcripts among the different samples for a particular treatment. The levels of one down-regulated gene, *CG4757*, are significantly different (p < 0.05) between the exposed and unexposed males.

## Authors' contributions

GEC conceived and executed the experiments, participated in the data analysis and wrote the manuscript.

## References

[B1] Sokolowski MB (2001). Drosophila: Genetics meets behavior. Nat Rev Genet.

[B2] Ben-Shahar Y, Robichon A, Sokolowski MB, Robinson GE (2002). Influence of gene action across different time scales on behavior. Science.

[B3] Ceriani MF, Hogenesch JB, Yanovsky M, Panda S, Straume M, Kay SA (2002). Genome-wide expression analysis in Drosophila reveals genes controlling circadian behavior. J Neurosci.

[B4] Toma DP, White KP, Hirsch J, Greenspan RJ (2002). Identification of genes involved in *Drosophila melanogaster *geotaxis, a complex behavioral trait. Nat Genet.

[B5] Anholt RRH, Dilda CL, Chang S, Fanara J-J, Kulkarni NH, Ganguly I, Rollmann SM, Kamdar KP, Mackay TFC (2003). The genetic architecture of odor-guided behavior in Drosophila: epistasis and the transcriptome. Nat Genet.

[B6] Dubnau J, Chiang A-S, Grady L, Barditch J, Gossweiler S, McNeil J, Smith P, Buldoc F, Scott R, Certa U, Broger C, Tully T (2003). The *staufen/pumilio *pathway is involved in Drosophila long-term memory. Curr Biol.

[B7] Grozinger CM, Sharabash NM, Whitfield CW, Robinson GE (2003). Pheromone-mediated gene expression in the honey bee brain. Proc Natl Acad Sci U S A.

[B8] Aubin-Horth N, Landry CR, Letcher BH, Hofmann HA (2005). Alternative life histories shape brain gene expression profiles in males of the same population. Proc R Soc B.

[B9] Mackay TFC, Heinsohn SL, Lyman RF, Moehring AJ, Morgan TJ, Rollmann SM (2005). Genetics and genomics of Drosophila mating behavior. Proc Natl Acad Sci USA.

[B10] Perrimon N, Mathey-Prevot B (2007). Applications of high-throughput RNA interface screens to problems in cell and developmental biology. Genetics.

[B11] Dietzl G, Chen D, Schnorrer F, Su K-C, Barinova Y, Fellner M, Gasser B, Kinsey K, Oppel S, Scheiblauer S, Couto A, Marra V, Keleman K, Dickson BJ (2007). A genome-wide transgenic RNAi library for conditional gene inactivation in Drosophila. Nature.

[B12] Greenspan RJ, Ferveur J-F (2000). Courtship in *Drosophila*. Ann Rev Genet.

[B13] Billeter J-C, Goodwin SF, O'Dell KMC (2002). Genes mediating sex-specific behaviors in *Drosophila*. Adv Genet.

[B14] Dukas R (2004). Male fruit flies learn to avoid interspecific courtship. Behav Ecol.

[B15] Cline TW, Meyer BJ (1996). Vive le difference: males vs females in flies vs worms. Ann Rev Genet.

[B16] Baker BS, Taylor BJ, Hall JC (2001). Are complex behaviors specified by dedicated regulatory genes? Reasoning from *Drosophila*. Cell.

[B17] Christiansen AE, Keisman EL, Ahmad SM, Baker BS (2002). Sex comes in from the cold: the integration of sex and pattern. Trends Genet.

[B18] Burtis KC, Coschigano KT, Baker BS, Wensink PC (1991). The Doublesex proteins of *Drosophila melanogaster *bind directly to a sex-specific yolk protein gene enhancer. EMBO J.

[B19] Cann MJ, Chung E, Levin R (2000). A new family of adenylyl cyclase genes in the male germline of *Drosophila melanogaster*. Dev Genes Evol.

[B20] Kopp IA, Carroll SB (2000). Genetic control and evolution of sexually dimorphic characters in *Drosophila*. Nature.

[B21] Dauwalder B, Tsujimoto S, Moss J, Mattox W (2002). The *Drosophila takeout *gene is regulated by the sex determination pathway and affects male courtship behavior. Genes Dev.

[B22] Fujii S, Amrein H (2002). Genes expressed in the *Drosophila *head reveal a role for fat cells in sex-specific physiology. EMBO J.

[B23] Bray S, Amrein H (2003). A putative Drosophila pheromone receptor expressed in male-specific taste neurons is required for efficient courtship. Neuron.

[B24] Drapeau MD, Radovic A, Wittkopp PJ, Long AD (2003). A gene necessary for normal male courtship, yellow, acts downstream of *fruitless *in the *Drosophila melanogaster *larval brain. J Neurobiol.

[B25] Arbeitman MN, Fleming AA, Siegal ML, Null BH, Baker BS (2004). A genomic analysis of *Drosophila *somatic sexual differentiation and its regulation. Development.

[B26] Demir E, Dickson BJ (2005). *fruitless *specifies male courtship behavior in *Drosophila*. Cell.

[B27] Kimura K-I, Ote M, Tazawa T, Yamamoto D (2005). Fruitless specifies sexually dimorphic neural circuitry in the Drosophila brain. Nature.

[B28] Manoli DS, Foss M, Villella A, Taylor BJ, Hall JC, Baker BS (2005). Male specific *fruitless *specifies the neural substrates of *Drosophila *courtship behaviour. Nature.

[B29] Stockinger P, Kvitsiani D, Rotkopf S, Tirian L, Dickson BJ (2005). Neural circuitry that governs *Drosophila *male courtship behavior. Cell.

[B30] Lawniczak MKN, Begun DJ (2004). A genome-wide analysis of courting and mating responses in *Drosophila melanogaster *females. Genome.

[B31] McGraw LA, Gibson G, Clark AG, Wolfner MF (2004). Genes regulated by mating, sperm, or seminal proteins in mated female *Drosophila melanogaster*. Curr Biol.

[B32] Rützler M, Zwiebel LJ (2005). Molecular biology of insect olfaction: recent progress and conceptual models. J Comp Physiol A Neuroethol Sens Neural Behav Physiol.

[B33] De Gregorio E, Spellman PT, Rubin GM, Lemaitre B (2001). Genome-wide analysis of the Drosophila immune response by using oligonucleotide microarrays. Proc Natl Acad Sci USA.

[B34] Irving P, Troxler L, Heuer TS, Kopczynski C, Reichert J-M, Hoffmann JA, Hetru C (2001). A genome-wide analysis of immune responses in Drosophila. Proc Natl Acad Sci USA.

[B35] Boutros M, Agaisse H, Perrimon N (2002). Sequential activation of signaling pathways during innate immune responses in Drosophila. Dev Cell.

[B36] De Gregorio E, Spellman PT, Tzou P, Rubin GM, Lemaitre B (2002). The Toll and Imd pathways are the major regulators of the immune response in Drosophila. EMBO J.

[B37] Park JM, Brady H, Ruocco MG, Sun H, Williams D, Lee SJ, Kato T, Richards N, Chan K, Mercurio F, Karin M, Wasserman SA (2004). Targeting of TAK1 by the NF-kappa B protein Relish regulates the JNK-mediated immune response in Drosophila. Genes Dev.

[B38] McKean KA, Nunney L (2001). Increased sexual activity reduces male immune function in *Drosophila melanogaster*. Proc Natl Acad Sci USA.

[B39] Radonjic M, Andrau J-C, Lijnzaad P, Kemmeren P, Kockelkorn TTJP, van Leenen D, van Berkum NL, Holstege FCP (2005). Genome-wide analyses reveal RNA polymerase II located upstream of genes poised for rapid response upon *S. cerevisiae *stationary phase exit. Mol Cell.

[B40] Burmeister SS, Jarvis ED, Fernald RD (2005). Rapid behavioral and genomic responses to social opportunity. PLoS Biol.

[B41] Irwin LN (2001). Gene expression in the hippocampus of behaviorally stimulated rats: analysis by DNA microarray. Molec Brain Res.

[B42] Bradley KC, Boulware MB, Jiang H, Doerge RW, Meisel RL, Memelstein PG (2005). Changes in gene expression in the nucleus accumbens and striatum following sexual experience. Genes Brain Behav.

[B43] Cavallaro S, Schreurs BG, Zhao W, D'Agata V, Alkon DL (2001). Gene expression profiles during long-term memory consolidation. Eur J Neurosci.

[B44] Cavallaro S, D'Agata V, Manickam P, Dufour F, Alkon DL (2002). Memory-specific temporal profiles of gene expression in the hippocampus. Proc Natl Acad Sci USA.

[B45] Saunders A, Core LJ, Lis JT (2006). Breaking barriers to transcription elongation. Nat Rev Mol Cell Biol.

[B46] Gilmour DS, Lis JT (1986). RNA Polymerase II interacts with the promoter region of the noninduced *hsp70 *gene in *Drosophila melanogaster *cells. Mol Cell Biol.

[B47] O'Brien T, Lis JT (1993). Rapid changes in Drosophila transcription after an instantaneous heat shock. Mol Cell Biol.

[B48] Hoffmann JA (2003). The immune response of Drosophila. Nature.

[B49] Holloway AK, Begun DJ (2004). Molecular evolution and population genetics of duplicated accessory gland protein genes in Drosophila. Mol Biol Evol.

[B50] Giot L, Bader JS, Brouwer C, Chaudhari A, Kuang B, Li Y, Hao YL, Ooi CE, Godwin B, Vitols E, Vijayadamodar G, Pochart P, Machineni H, Welsh M, Kong Y, Zerhusen B, Malcolm R, Varrone Z, Collis A, Minto M, Burgess S, McDaniel L, Stimpson E, Spriggs F, Williams J, Neurath K, Ioime N, Agee M, Voss E, Furtak K, Renzulli R, Aanensen N, Carrolla S, Bickelhaupt E, Lazovatsky Y, DaSilva A, Zhong J, Stanyon CA, Finley RL, White KP, Braverman M, Jarvie T, Gold S, Leach M, Knight J, Shimkets RA, McKenna MP, Chant J, Rothberg JM (2003). A protein interaction map of *Drosophila melanogaster*. Science.

[B51] Chintapalli VR, Wang J, Dow JAT (2007). Using FlyAtlas to identify better Drosophila models of human disease. Nat Genet.

[B52] Erdman SE, Burtis KC (1993). The *Drosophila *doublesex proteins share a novel zinc finger related DNA binding domain. EMBO J.

[B53] Ryner LC, Goodwin SF, Castrillon DH, Anand A, Villella A, Baker BS, Hall JC, Taylor BJ, Wasserman SA (1996). Control of male sexual behavior and sexual orientation in *Drosophila *by the *fruitless *gene. Cell.

[B54] Billeter J-C, Villella A, Allendorfer JB, Dornan AJ, Richardson M, Gailey DA, Goodwin SA (2006). Isoform-specific control of male neuronal differentiation and behavior in Drosophila by the *fruitless *gene. Curr Biol.

[B55] Galindo K, Smith DP (2001). A large family of divergent Drosophila odorant-binding proteins expressed in gustatory and olfactory sensilla. Genetics.

[B56] Boutanaev AM, Kalmykova AI, Shevelyov, Nurminsky DI (2002). Large clusters of co-expressed genes in the Drosophila genome. Nature.

[B57] Li J, Wong L (2001). Emerging patterns and gene expression data. Genome Inform.

[B58] Baldi P, Long AD (2001). A Bayesian framework for the analysis of microarray expression data: regularized t-test and statistical inferences of gene changes. Bioinformatics.

[B59] Carney GE, Taylor BJ (2003). *logjam *encodes a predicted EMP24/GP25 protein that is required for *Drosophila *oviposition behavior. Genetics.

